# Longitudinal Adherence to Screening for Colorectal, Cervical, and Lung Cancer in a US Consortium

**DOI:** 10.1007/s11606-025-09835-6

**Published:** 2025-10-07

**Authors:** Ethan A. Halm, Natalie J. Del Vecchio, Katharine A. Rendle, Jasmin A. Tiro, Yingye Zheng, Rachel L. Winer, Jennifer S. Haas, Douglas A. Corley, Celette Sugg Skinner, Joanne Schottinger, Nirupa R. Ghai, Jessica Chubak

**Affiliations:** 1 Department of Medicine, Rutgers Robert Wood Johnson Medical School, 112 Paterson Street, New Brunswick, NJ 08901 USA; 2 Public Health Sciences Division, Fred Hutchinson Cancer Center, Seattle, WA USA; 3 Department of Family Medicine & Community Health, Perelman School of Medicine, University of Pennsylvania, Philadelphia, PA USA; 4 Department of Public Health Sciences, University of Chicago Biological Sciences Division, Chicago, IL USA; 5 Department of Epidemiology, University of Washington School of Public Health, Seattle, WA USA; 6 Division of General Internal Medicine, Massachusetts General Hospital, Boston, MA USA; 7 Division of Research, Kaiser Permanente Northern California, Pleasanton, CA USA; 8 Peter O’Donnell Jr. School of Public Health and Simmons Comprehensive Cancer Center, University of Texas Southwestern Medical Center, Dallas, TX USA; 9 Department of Research & Evaluation, Kaiser Permanente Bernard J. Tyson School of Medicine, Pasadena, CA USA; 10 Investigative Science Division, Kaiser Permanente Washington Health Research Institute, Seattle, WA USA

**Keywords:** colorectal cancer, cervical cancer, lung cancer, screening adherence

## Abstract

**Background:**

Effective screening for colorectal, cervical, and lung cancer requires adherence over time, but little is known about repeat testing in real-world practice.

**Objective:**

Describe patterns of longitudinal screening adherence and identify patient and system factors associated with repeat testing.

**Design:**

Retrospective cohort study of colorectal, cervical, or lung cancer screening in 2010–2019.

**Participants:**

Adults eligible for repeat colorectal (stool-based), cervical, or lung cancer screening following a negative index test in ten regional health systems comprising the US PROSPR consortium.

**Main Measures:**

Repeat screening based on guideline-recommended intervals. For the colorectal and lung cohorts with opportunities for multiple annual screening rounds, the main outcome was repeat screening categorized as none, inconsistent, or consistent.

**Results:**

The sample size was: 1,566,346 for colorectal, 216,344 for cervical, and 6,209 for lung cancer screening. For colorectal, cervical, and lung screeners, mean age at index was 58.2, 39.4, and 64.6 years, respectively, and 49%, 55% and 30% were Hispanic and/or non-white. Completion of the next screening round was 62% for colorectal, 56% for cervical, and 56% for lung cancer. For colorectal, over the next two rounds of testing, 53% were consistent, 33% inconsistent, and 14% no repeat screeners. The comparable percentages over 3 + rounds for colorectal were 40% consistent, 50% inconsistent, and 11% no repeat screeners. For lung, over the next two rounds, 47% were consistent, 31% inconsistent, and 22% no repeat screeners. The proportions over 3 + rounds for lung were 44% consistent, 42% inconsistent, and 14% no repeat screening. The health system was the strongest predictor of repeat and consistent testing with three- to ten-fold variation.

**Conclusions:**

Adherence to longitudinal screening for colorectal, cervical and lung cancer was suboptimal, particularly as the number of testing rounds increased. System-level strategies are needed to increase screening adherence given the strong relationship between health system and outcomes.

**Supplementary Information:**

The online version contains supplementary material available at 10.1007/s11606-025-09835-6.

## INTRODUCTION

Cancer screening, if done optimally, requires repeated adherence over time rather than one-time testing. The US Preventive Services Task Force (USPSTF) recommends regular screening to detect colorectal, cervical and lung cancer early because screening effectiveness improves when aligned with recommended intervals.^1–3^ National statistics generally report prevalence of being screen up-to-date at one point in time.^4^ There is less research on the consistency of screening over time. When examined, rates of repeat testing are much lower than the screening adherence under optimal conditions assumed by guideline developers and those modeling the comparative effectiveness of different screening strategies.^1–3^ Most studies of repeat testing have focused on one type of cancer screening, over short time periods, and often in one health system. Much less is known about whether patterns of repeat testing across multiple cancer types are similar or different, and whether there are a common set of patient and system factors associated with longitudinal screening adherence.

This study sought to characterize patterns and predictors of repeat screening for colorectal, cervical, and lung cancer in real-world practices among diverse populations using data from a large U.S. cancer screening research consortium (Population-Based Research to Optimize the Screening Process (PROSPR II).^5,6^ Our aims were to: 1) Describe the proportion of those who: did not repeat screening, were inconsistent screeners, and consistent screeners, and 2) Identify patient and system factors associated with repeat screening. We report on: 1) “Next round” screening for all three cancer cohorts (colorectal [stool-based], lung, and cervical) with at least one round of screening eligibility after a negative test; and, 2) “Annual screening consistency” for colorectal and lung cancer screening patients with a negative result on an annual test (home stool test or chest CT) with multiple rounds of screening eligibility.

## METHODS

### Study Setting & Data Collection

Ten regional health systems comprise the National Cancer Institute’s PROSPR II consortium.^5,6^ We included 2010–2019 data from the colorectal cancer (CRC) screening sites of Kaiser Permanente Northern California (KPNC), Kaiser Permanente Southern California (KPSC), Kaiser Permanente Washington (KPWA), and Parkland Health (PH). The cervical cancer screening data were from 2010–2019 at KPWA, PH, and Mass General Brigham (MGB). The lung cancer screening data were from 2010–2019 at Kaiser Permanente Colorado, University of Pennsylvania Health System, Henry Ford Health, Kaiser Permanente Hawaii, and Marshfield Clinic Research Institute. Sociodemographic and clinical characteristics, screening tests, and primary care visits were derived from electronic health record (EHR) and administrative data.

### Human Ethics and Consent to Participate

PROSPR II was IRB-approved at all sites.

### Study Population

This retrospective, longitudinal cohort study focused on all adults eligible for guideline-recommended screening at participating sites who had a negative test during 2010–2018 for CRC, 2010–2016 for cervical, and 2014–2018 for lung. The first negative test during this period was considered the “index” screen. Those ineligible for screening based on age, history of the cancer or other high-risk conditions were excluded. See eTable 1 for details on the inclusion/exclusion criteria by cancer type. The CRC screening cohort included adults 50–75 years with a negative index fecal test (immunohistochemical test [FIT] or occult blood test [FOBT]), no prior colorectal cancer, adenoma, colectomy, proctectomy, inflammatory bowel disease, colonoscopy in 10 years, or sigmoidoscopy in 5 years. We focused on stool-based tests and not endoscopy-based screening due to endoscopy’s extended length of screening coverage. We excluded individuals with a premature repeat test < 9 months after index screen (FIT/FOBT, colonoscopy, sigmoidoscopy, colonography), and those with a new adenoma/cancer < 9 months as these events precluded on-time repeat screening (eFigure 1).

The cervical cancer screening cohort included adults 21–65 years with a negative index Pap test during 2010–2016 (and ≥ 39 months of observation time) or HPV co-test in 2010–2014 (and ≥ 63 months of observation time) and no history of hysterectomy, HIV, cervical abnormality or cancer. Individuals with an early re-test (< 9 months after index) or with an abnormal screen, colposcopy, treatment, or cervical cancer) were excluded (eFigure 2).

The lung cancer screening cohort included adults 55–80 years with a history of smoking, a negative index low dose computed tomography (LDCT), and no history of lung cancer. Individuals with early LDCT or other chest CT (< 9 months after index screen) or lung cancer were excluded (eFigure 3). Given missingness of pack-years and quit-year in the EHR even among those with completed screening, this information was not required for patients to be included in this analysis, an approach used in prior studies.^7^

### Screening Outcomes and Covariates

Patients became ineligible for analysis of repeat rounds of screening at the time of a subsequent positive test, developing cancer, disengagement from the health system, death, target organ removal, or completing another screening modality obviating the need for screening in the next interval. The analyses of “next round screening” examined all three cohorts with a negative index test and sufficient study observation time to complete the next interval screen (15 months for CRC and lung, 39 or 63 months for cervical cancer depending on the index test). We added three months to each recommended testing round as leeway in real-world practice, a convention used in prior studies.^7,8^ The “Annual Screening Consistency” analyses used the colorectal and lung cancer cohorts with sufficient study observation time for two or more rounds of repeat screening (30–44 months for two and 45–60 months for three tests). The much longer screening interval for cervical cancer (3–5 years) precluded analyses of screening consistency over multiple rounds.

The “Next round screening” outcome was repeating a test within 9–15 months (CRC and lung) or 9–39 months (cervical). “Annual screening consistency” was defined as: 1)"Consistent,” 2/2 or 3/3 subsequent rounds completed; 2) “Inconsistent,” 1/2, 1/3, 2/3 rounds completed, and 3) “No Repeat Screening,” 0/2 or 0/3 completed rounds. Analyses were stratified by study observation time: those with two screening rounds of observation time (30–44 months of follow-up) or three or more screening rounds (45 + months of follow-up). See eFigures 4 and 5 for details.

For the “Next Screening” analyses in CRC, we counted any potential screening test (FIT/FOBT, colonoscopy, sigmoidoscopy, barium enema, CT colonography, or lower endoscopy) after the index stool test. Those with colonoscopy or sigmoidoscopy as second test were excluded from the “Annual Screening Consistency” analyses (which examined annual testing). Repeat lung cancer screening was defined as any chest CT after index LDCT. For cervical cancer, repeat screening was defined as a Pap test or co-testing (Pap and HPV test). For all cohorts, we counted all subsequent tests regardless of indication as this aligns with usual clinical practice and quality assessment definitions of being screen up-to-date.

Patient-level characteristics included: age, sex, race/ethnicity, insurance, and Charlson Comorbidity Score, body mass index, and smoking status in the calendar year of the index test. Primary care visits were from the year prior to the index screen. The healthcare organization where the patient was enrolled or connected to primary care was the main system variable. The health systems and their associated cancer screening strategies are described in Table 4.

### Statistical Analysis

We used descriptive statistics to characterize the patients in each organ and subgroup. Adjusted odds ratios (AORs) and 95% confidence intervals from multivariable logistic regression were used to examine if patient, visit, and system characteristics were associated with next round repeat screening. Multinomial logistic regression was used to assess correlates of annual screening consistency (consistent, inconsistent, or no repeat screening). All covariates in multivariable models were determined a priori due to hypothesized associations with screening. Health system impact was modeled as a fixed effect. Analyses were conducted using SAS 9.4 (SAS Institute, Cary, NC).

## RESULTS

Characteristics of the CRC (N = 1,566,347), cervical cancer (N = 216,344) and lung cancer (N = 6,209) repeat screening-eligible cohorts are shown in eTable 2. For colorectal, cervical, and lung screeners, mean age at index was 58.2, 39.4, and 64.6 years, respectively, and 49%, 55% and 30% were Hispanic and/or non-white. The ages, sex, race/ethnicity, insurance status, and comorbid illness burden distributions varied considerably by organ type and the health systems represented in each cohort.

### Next Round Screening Adherence

**Figure 1:**
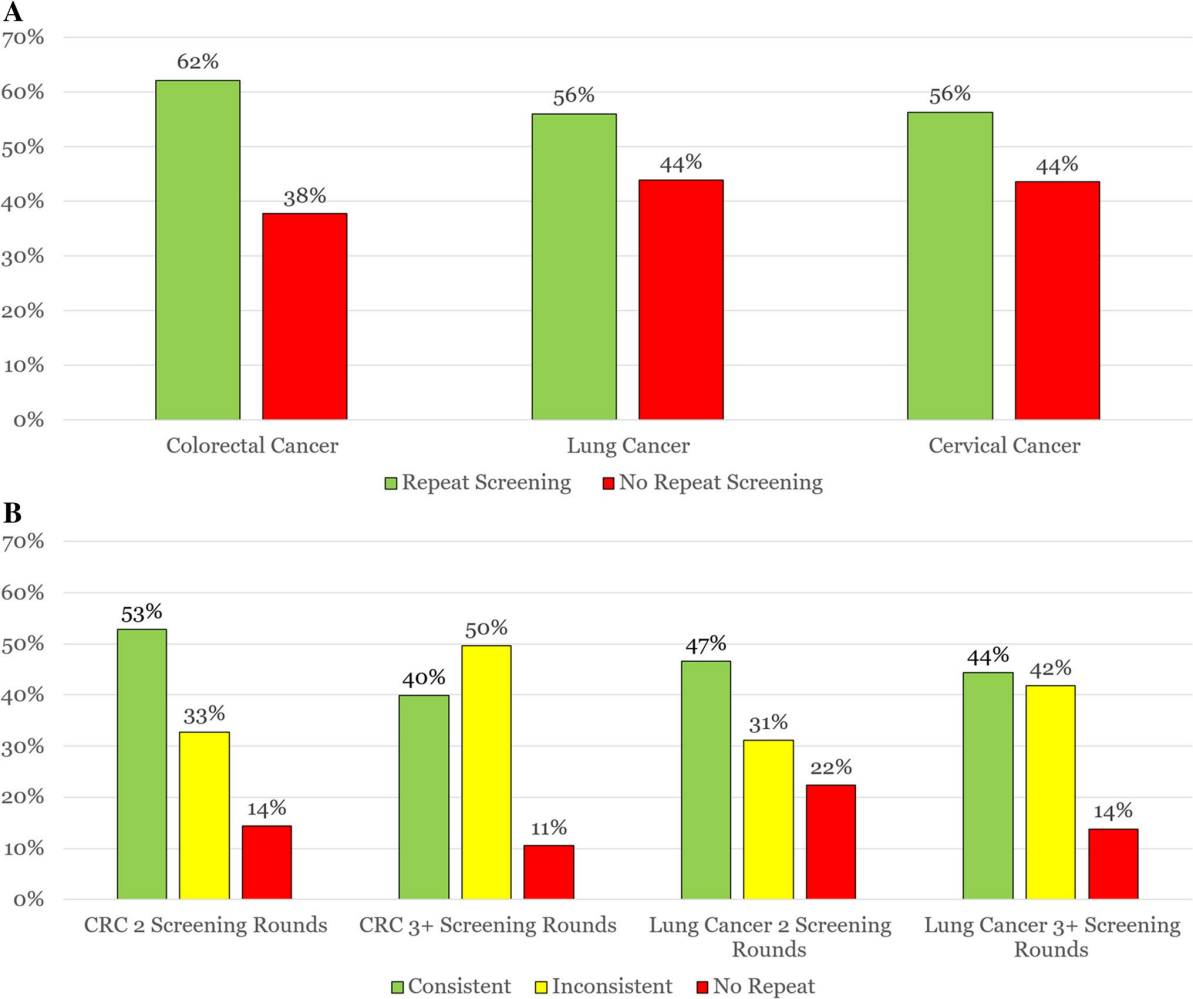
A Patterns of Next Round Repeat Screening by Cancer Type. B Patterns of Annual Screening Consistency in the Colorectal and Lung Cancer Screening Cohorts Stratified by Number of Screening Rounds. CRC: Colorectal cancer; Fig. 1B shows screening consistency results for CRC and lung cancer screening stratified by study observation time categories defined as: 2 screening rounds of study observation time: 30–44 months of follow-up time after negative index test; 3 + screening rounds of study observation time: 45 + months of follow-up time after negative index test. Consistent screening was completing 2/2 or 3/3 rounds and inconsistent screening was completing 1/2, 1/3, 2/3 rounds.

Across all three cancer types, receipt of the next round of screening was suboptimal with only 62% of patients repeating their next CRC test (site variation: 19%−71%) and 56% of patients completing their next cervical (site variation: 45%−67%) and lung cancer (site variation: 31%−86%) test (Fig. 1A).

### Annual Screening Consistency Adherence

The consistency of screening over multiple subsequent rounds of observation time for CRC and lung cancer is shown in Fig. 1B. For colorectal, over the next two rounds for testing, 53% were consistent, 33% inconsistent, and 14% no repeat screeners. The comparable percentages over 3 + rounds for colorectal were 40% consistent, 50% inconsistent, and 11% no repeat screeners. For lung, over the next two potential testing rounds, 47% were consistent, 31% inconsistent, and 22% no repeat screeners. The proportions over 3 + rounds for lung were 44% consistent, 42% inconsistent, and 14% no repeat screening.

### Predictors of Repeating the Next Round of CRC Screening

The health system site was the strongest predictor of repeating the next CRC screen with nearly ten-fold variation in the adjusted odds (Table 1). Patients who were younger, female, had BMI ≥ 25 or < 18.5, a comorbidity score ≥ 3, and smoked had lower odds of repeat screening compared to their counterparts. Patients who were non-white were less likely to repeat screening than those who were non-Hispanic white (but not Asian). Those with government-funded insurance and without a PCP visit in the prior year had higher odds of repeating.

**Table 1:** Factors Associated with Repeating the Next Round of Colorectal Cancer Screening

Characteristic	Repeat screening n = 852,230 (62.2%)	No repeat screening n = 516,819 (37.8%)	Adjusted odds ratio for repeat screening (95% CI)
Health system site			
A	513,562 (60.3%)	214,123 (41.4%)	1.00 (REF)
B	17,544 (2.1%)	36,142 (7.0%)	0.20 (0.20–0.20)
C	3741 (0.4%)	15,502 (3.0%)	0.11 (0.10–0.11)
D	317,383 (37.2%)	251,052 (48.6%)	0.64 (0.63–0.64)
Age (years)	58.4 (6.9)	57.5 (6.3)	1.01 (1.01–1.01)
Sex			
Male	397,859 (46.7%)	235,807 (45.6%)	1.00 (REF)
Female	454,371 (53.3%)	281,012 (54.4%)	0.94 (0.93–0.95)
Race/Ethnicity^a^			
White	455,623 (53.5%)	254,496 (49.2%)	1.00 (REF)
Black	58,756 (6.9%)	44,265 (8.6%)	0.88 (0.86–0.89)
Asian	132,117 (15.5%)	61,847 (12.0%)	1.11 (1.09–1.12)
Native Hawaiian/Pacific Islander	5034 (0.6%)	3512 (0.7%)	0.83 (0.79–0.87)
American Indian/Alaskan Native	2326 (0.3%)	1697 (0.3%)	0.80 (0.75–0.86)
Hispanic	167,092 (19.6%)	127,915 (24.8%)	0.86 (0.85–0.87)
Multiple/another	31,282 (3.7%)	23,087 (4.5%)	0.82 (0.80–0.83)
Body mass index (BMI)			
BMI ≥ 18.5—< 25	168,581 (19.8%)	73,312 (14.2%)	1.00 (REF)
BMI ≥ 25—< 30	214,011 (25.1%)	107,868 (20.9%)	0.89 (0.88–0.90)
BMI ≥ 30	186,790 (21.9%)	113,792 (22.0%)	0.79 (0.78–0.80)
BMI < 18.5	5088 (0.6%)	2335 (0.5%)	0.91 (0.87–0.96)
Unknown	277,760 (32.6%)	219,512 (42.5%)	0.75 (0.74–0.76)
Charlson comorbidity score			
0	615,276 (72.2%)	367,645 (71.1%)	1.00 (REF)
1	133,025 (15.6%)	84,155 (16.3%)	0.99 (0.98–1.00)
2	60,001 (7.0%)	36,590 (7.1%)	1.00 (0.98–1.01)
≥ 3	439,28 (5.2%)	28,429 (5.5%)	0.89 (0.88–0.91)
Smoking status			
Never smoked or unknown	642,110 (75.3%)	369,766 (71.5%)	1.00 (REF)
Ever smoked	210,120 (24.7%)	147,053 (28.5%)	0.87 (0.86–0.88)
Insurance			
Commercial/private	631,662 (74.1%)	407,056 (78.8%)	1.00 (REF)
Medicaid	13,692 (1.6%)	10,462 (2.0%)	1.09 (1.06–1.13)
Medicare	201,943 (23.7%)	88,266 (17.1%)	1.39 (1.37–1.40)
Other government or uninsured	4933 (0.6%)	11,035 (2.1%)	1.19 (1.13–1.25)
PCP visit in year prior to index			
Yes	691,424 (81.1%)	427,122 (82.6%)	1.00 (REF)
No	160,806 (18.9%)	89,697 (17.4%)	1.02 (1.01–1.03)

^a^All race/ethnic categories are non-Hispanic except those included in the Hispanic category. “Multiple” race includes those with more than one race listed. The adjusted models included all variables in the table. Age, insurance, Charlson score, smoking status, and BMI were from the year of index screen, with BMI also using last result carried-forward up to 2 years (due to larger quantity of missing data). “Commercial/Private” includes commercial or high deductible insurance. “Other Government” insurance includes publicly funded medical assistance programs that are not Medicare or Medicaid

### Predictors of Repeating the Next Round of Cervical Cancer Screening

There was three-fold variation by site in the adjusted odds of repeating the next cervical cancer screening test (Table 2). Other factors associated with lower odds of repeat screening included: older age; BMI ≥ 25 or < 18.5; being Asian or Native Hawaiian/Pacific Islander; smoking; Medicaid or Medicare insurance; and no PCP visit in the prior year. Hispanic ethnicity, higher comorbidity, and being uninsured or covered by other government programs was associated with higher odds of repeating.

**Table 2:** Factors Associated with Repeating the Next Round of Cervical Cancer Screening

Characteristic	Repeat screening n = 114,910 (56.4%)	No repeat screening n = 88,874 (43.6%)	Adjusted odds ratio for repeat screening (95% CI)
Health system site			
A	35,109 (30.6%)	42,099 (47.4%)	1.00 (REF)
B	29,967 (26.1%)	22,655 (25.5%)	1.97 (1.88–2.05)
C	49,834 (43.4%)	24,120 (27.1%)	3.03 (2.92–3.14)
Age (years)	39.4 (11.7)	39.5 (12.3)	0.99 (0.99–0.99)
Race/ethnicity^a^			
White	54,989 (47.9%)	34,135 (38.4%)	1.00 (REF)
Black	11,293 (9.8%)	12,617 (14.2%)	0.99 (0.96–1.03)
Asian	7782 (6.8%)	6016 (6.8%)	0.88 (0.85–0.91)
Native Hawaiian/Pacific Islander	324 (0.3%)	342 (0.4%)	0.79 (0.67–0.92)
American Indian/Alaskan Native	278 (0.2%)	249 (0.3%)	0.92 (0.77–1.09)
Hispanic	36,913 (32.1%)	33,012 (37.1%)	1.41 (1.37–1.46)
Multiple/another	3331 (2.9%)	2503 (2.8%)	0.86 (0.81–0.90)
Body mass index (BMI)			
BMI ≥ 18.5—< 25	42,585 (37.1%)	25,653 (28.9%)	1.00 (REF)
BMI ≥ 25—< 30	34,236 (29.8%)	26,106 (29.4%)	0.92 (0.90–0.95)
BMI ≥ 30	36,278 (31.6%)	35,895 (40.4%)	0.77 (0.75–0.78)
BMI < 18.5	1811 (1.6%)	1220 (1.4%)	0.85 (0.79–0.92)
Charlson comorbidity score			
0	97,024 (84.4%)	75,033 (84.4%)	1.00 (REF)
1	12,072 (10.5%)	9,454 (10.6%)	1.05 (1.02–1.08)
2	4147 (3.6%)	2952 (3.3%)	1.12 (1.07–1.18)
≥ 3	1667 (1.5%)	1435 (1.6%)	1.18 (1.09–1.27)
Smoking status			
Never smoked or unknown	89,063 (77.5%)	67,280 (75.7%)	1.00 (REF)
Ever smoked	25,847 (22.5%)	21,594 (24.3%)	0.87 (0.85–0.88)
Insurance			
Commercial/private	68,023 (59.2%)	40,576 (45.7%)	1.00 (REF)
Medicaid	23,612 (20.6%)	24,558 (27.6%)	0.73 (0.71–0.76)
Medicare	1836 (1.6%)	1736 (2.0%)	0.80 (0.74–0.86)
Other government or uninsured	21,439 (18.7%)	22,004 (24.8%)	1.07 (1.02–1.11)
PCP visit year prior to index			
Yes	63,206 (55.0%)	45,600 (51.3%)	1.00 (REF)
No or unknown	51,704 (45.0%)	43,274 (48.7%)	0.97 (0.96–0.99)

^a^All race/ethnic categories are non-Hispanic except those included in the Hispanic category. AI = American Indian, and AN = Alaskan Native. “Multiple” race includes those with more than one race listed. The adjusted models included all variables in the table. Age, insurance, Charlson score, smoking status, and BMI were from the year of index screen, with BMI also using last result carried-forward up to 2 years. “Commercial/Private” includes commercial or high deductible insurance. “Other Government” insurance includes publicly funded programs that cover cervical cancer screening such as family planning grants and the National Breast and Cervical Cancer Early Detection Program, as well as county medical assistance programs

### Predictors of Repeating the Next Round of Lung Cancer Screening

There was ten-fold variation by health system in the adjusted odds of repeating the next lung cancer screen (Table 3). Patients who were younger, Black or Hispanic, and currently smoking had lower odds of repeating. Those with 1–2 comorbidities or no PCP visit in the prior year had higher odds of repeating.

**Table 3:** Factors Associated with Repeating the Next Round of Lung Cancer Screening

Characteristic	Repeat screening n = 3306 (56.1%)	No repeat screening n = 2586 (43.9%)	Adjusted odds ratio for repeat screening (95% CI)
Health system site			
A	1635 (49.5%)	505 (19.5%)	1.00 (REF)
B	420 (12.7%)	68 (2.6%)	2.08 (1.50–2.87)
C	690 (20.9%)	1177 (45.5%)	0.20 (0.17–0.23)
D	277 (8.4%)	190 (7.4%)	0.45 (0.36–0.56)
E	284 (8.6%)	646 (25.0%)	0.15 (0.13–0.19)
Age (years)	65.3 (5.8)	63.9 (5.7)	1.02 (1.01–1.04)
Sex			
Male	1803 (54.5%)	1369 (52.9%)	1.00 (REF)
Female	1503 (45.5%)	1217 (47.1%)	1.04 (0.92–1.17)
Race/Ethnicity^a^			
White	2440 (73.8%)	1673 (64.7%)	1.00 (REF)
Black	287 (8.7%)	618 (23.9%)	0.68 (0.57–0.81)
Asian	176 (5.3%)	46 (1.8%)	0.97 (0.64–1.47)
Native Hawaiian/Pacific Islander	19 (0.6%)	9 (0.4%)	0.44 (0.18–1.08)
American Indian/Alaskan Native	10 (0.3%)	< 6	1.38 (0.44–4.35)
Hispanic	144 (4.4%)	77 (3.0%)	0.71 (0.52–0.96)
Multiple/another	230 (7.0%)	158 (6.1%)	0.80 (0.63–1.03)
Body mass index (BMI)			
BMI ≥ 18.5—< 25	817 (24.7%)	612 (23.7%)	1.00 (REF)
BMI ≥ 25—< 30	1227 (37.1%)	880 (34.0%)	1.10 (0.95–1.29)
BMI ≥ 30	1194 (36.1%)	1031 (39.9%)	1.01 (0.86–1.18)
BMI < 18.5	68 (2.1%)	63 (2.4%)	0.88 (0.59–1.32)
Charlson comorbidity score			
0	1073 (32.5%)	1001 (38.7%)	1.00 (REF)
1	1058 (32.0%)	789 (30.5%)	1.22 (1.06–1.41)
2	517 (15.6%)	362 (14.0%)	1.30 (1.08–1.56)
≥ 3	658 (19.9%)	434 (16.8%)	1.12 (0.94–1.33)
Smoking status			
Former smoker	1689 (51.1%)	979 (37.9%)	1.00 (REF)
Current smoker	1617 (48.9%)	1607 (62.1%)	0.68 (0.60–0.76)
Insurance			
Commercial/private	1131 (34.2%)	1078 (41.7%)	1.00 (REF)
Medicaid	258 (7.8%)	295 (11.4%)	0.82 (0.66–1.02)
Medicare	1913 (57.9%)	1207 (46.7%)	0.97 (0.83–1.14)
Other government or uninsured	< 6	6 (0.2%)	1.08 (0.30–3.91)
PCP visit year prior to index			
Yes	3196 (96.7%)	2512 (97.1%)	1.00 (REF)
No	110 (3.3%)	74 (2.9%)	1.52 (1.10–2.10)

^a^All race/ethnic categories are non-Hispanic except those included in the Hispanic category. “Multiple” race includes those with more than one race listed. The adjusted models included all variables in the table. Age, insurance, Charlson score, smoking status, and BMI were from the year of index screen, with smoking status and BMI also using last result carried-forward up to 2 years. “Commercial/Private” includes commercial or high deductible insurance. “Other Government” insurance includes publicly funded medical assistance programs that are not Medicare or Medicaid. Very small cell sizes are reported as < 6 with no % as per LOTUS PROSPR policy

### Predictors of Annual Screening Consistency for CRC Over Multiple Rounds

Health system was the strongest predictor of longer-term annual screening consistency for CRC (eTable 3). Patients who were Black, Native Hawaiian/Pacific Islander, American Indian/Alaska Native, or Hispanic, history of smoking, or had higher BMI or comorbidity burden were less likely to be consistent screeners. Those who were Asian, had Medicare or other government-funded insurance, or no PCP visit in the prior had higher odds of consistent testing.

### Predictors of Annual Screening Consistency for Lung Cancer Over Multiple Rounds

Large variation at the health system level was the most notable finding regarding screening consistency for lung cancer (eTable 4). Higher comorbidity (score = 1–2) and not having a PCP visit in the prior year was associated with lower odds of consistent screening in the largest subgroup of patients with 30–44 months of observation time.

## DISCUSSION

This study examining patterns of longitudinal adherence with screening for colorectal, lung, and cervical cancer across 10 large regional health systems, found that repeat testing in real-world practice was suboptimal and lower than the consistency of adherence assumed by national guidelines and policy simulation models.^1–3^ Across all three cancers, not much more than half of individuals completed a screening test in the next recommended interval (62% for CRC, and 56% for lung and cervical cancer). Adherence over subsequent rounds declined further. For CRC, only 53% and 40% were consistent screeners over the next two and 3 + rounds respectively. For lung cancer, less than half were consistent screeners (47% and 44% of those with two and 3 + rounds). Of greatest concern, for CRC and lung cancer screening between one in ten and one in five patients did not repeat *any* tests over the next two or more rounds for screening modalities intended to be done annually (11–14% for CRC and 14–22% for lung).

These findings advance the literature on screening adherence as most prior work examined up-to-date status at one point in time, in single cancers, or one health system. A study of repeating CRC screening from an earlier period in PROSPR found that among patients expected to do annual FIT testing, 47% had consistent screening, 43% inconsistent screening, and 10% no repeat screening over three years of follow-up.^8^ For lung cancer, a large community health system study reported that 37% of patients repeated a CT within 15 months after a low-risk scan, and only 51% repeated screening in the next five years.^9^ Others reported suboptimal repeat cervical cancer screening varying from 20 to 40%.^10,11^

Our most striking finding was major differences in repeat screening across the health systems (19%−71% for CRC, 45%−67% for cervical, and 31–86% for lung) amounting to three- to ten-fold variation by site in adjusted odds of adherence. Organizational impact is often unexamined because most prior studies were in single healthcare systems or a community-based samples from multiple healthcare systems that could not assess system-level effects. While PROSPR has reported strong site associations with cancer-specific screening over shorter time periods,^7,8,12^ seeing this theme confirmed across three types of cancer and longer time frames is notable. In general, the health systems in PROSPR with more centrally organized, population health-oriented screening outreach programs had higher rates of adherence (Table 4).^7,8,12–14^

**Table 4:** Characteristics and Screening Strategies of Healthcare Systems in the PROSPR Consortium

Health system sites	Type of system	Geographic region	Screening strategy
Colorectal cancer screening consortium			
A	Managed care integrated regional health system	West	Organized program plus opportunistic screening, outreach with mailed FIT, FIT also available at labs and other clinics, mailed letter on 50th birthday or anniversary of prior screening, EHR reminders if due/overdue
B	Mixed model regional health system	West	Organized program plus opportunistic screening, outreach strategies varied over time but included mailed reminder letters on birthday or anniversary of prior screening, EHR reminders if due/overdue
C	Integrated county safety net health system	South	Opportunistic screening 2010–2016, organized screening added 2017–2018 with mailed FIT if overdue, EHR reminders if due/overdue
D	Managed care integrated regional health system	West	Organized program plus opportunistic screening, outreach varied over time but included mailed FIT, mailed letter on 50th birthday or anniversary of prior screening, EHR reminders if due/overdue
Cervical cancer screening consortium			
A	Integrated county safety net health system	South	Opportunistic screening, eligible women are screened during clinic visits, screening performed by specialty and primary care, EHR reminders if due/overdue
B	Mixed model regional health system	West	Organized program plus opportunistic screening, birthday letter reminders to eligible women from population health team. Telephone outreach by primary care if overdue, EHR reminders if due/overdue
C	Integrated regional health system	Northeast	Opportunistic screening, eligible women are screened during clinic visits, EHR reminders if due/overdue
Lung cancer screening consortium			
A	Managed care regional health system	West	Organized program plus opportunistic screening, centralized assessment, ordering provider documents eligibility, provider referral to navigator who places order for LDCT
B	Managed care regional health system	West	Opportunistic screening, provider-driven assessment, navigator confirms eligibility at order, provider referral to navigator who places order for LDCT
C	Mixed model regional healthcare system	Midwest	Organized program plus opportunistic screening, centralized assessment, ordering provider documents eligibility at order, primary care or specialty care provider orders LDCT
D	Mixed model regional healthcare system	Midwest	Opportunistic screening, provider-driven assessment, ordering provider documents eligibility, primary care or specialty care provider orders LDCT
E	Mixed model regional healthcare system	Northeast	Opportunistic screening, provider-driven assessment, navigator confirms eligibility at scheduling, primary care or specialty care provider orders LDCT

Type of system: Managed care were predominantly HMO/health plan enrolled populations (commercial and public); Mixed model included both managed care contract and fee-for-service populations

Region: based on the four US Census Bureau geographic areas

Screening strategy: Organized screening programs employed system-wide, centralized strategies to identify and reach out to due/overdue patients in addition to opportunistic, visit-based screening; Opportunistic refers to primarily visit-based screening

This Table summarizes the gist of the key components of screening strategies used by each health system over the study period. Different program elements were implemented and scaled over time to varying degrees

Given the different cancer types, testing regimens, and underlying patient populations studied, we expected that associations between repeat screening and patient factors would vary across the cohorts. However, some commonalities were observed. People who had smoked (cervical and CRC) and current smokers (lung) were less likely to repeat testing consistent with studies associating tobacco use with lower screen up-to-date rates at one point in time.^15^ Older age was associated with more repeat screening for CRC and lung cancer, though the opposite was seen in cervical cancer.^16^ This may be because younger women are more likely to receive obstetrical/gynecological care for contraception and pregnancy. Additionally, older women may transition to co-testing with longer follow-up intervals, have lower perceived risk, or have more discomfort with pelvic exams. In contrast, CRC and lung cancer screening starts in middle age and continues as risk increases with age. Black and Hispanic adults had lower odds of repeat testing for CRC and lung cancer, congruent with known disparities in initial screening uptake potentially due to differences in non-access factors like health beliefs, literacy, or mistrust.^17,18^ Hispanic individuals had more repeat testing for cervical cancer, which differs from other reports perhaps because uninsurance in the health systems we studied were lower than the national average. Many Hispanic patients in the safety net health systems in PROSPR were often covered by family planning block grants.^19^ Being overweight was associated with lower odds of repeat testing in CRC and cervical cancer in other studies as well.^8,20^ The association between screening and a PCP visit in the prior year varied by organ type, possibly due to differences in who orders and performs testing for each cancer. For cervical cancer screening, gynecologists are PCPs and many internal and family medicine providers perform Pap smears. We are not sure why we saw the opposite PCP association for CRC and lung cancer. This might be because many patients had home stool testing or CTs ordered by organized population health outreach programs or specialists, not PCPs.^7,21^ Individuals without a recent PCP visit could potentially be healthier in ways not captured by the Charlson comorbidity score.

Several implications for practice and policy are worth noting. Providers looking to improve screening adherence over time should emulate the population health-oriented programs shown to increase screening.^8,13,14,21–25^ This includes centralized screening programs with tracking and accountability, outreach strategies that proactively contact patients who are not up-to-date (and do not rely solely on visit-based screening), use home-based testing when available, and patient navigation (eTable2). Since many patients do not repeat screening as frequently as recommended even in the highest performing health systems, researchers and public health professionals will need to develop additional strategies to further improve longitudinal adherence.

From a policy perspective, national guideline groups should recommend strategies known to maximize repeat screening. Public and private cancer-focused organizations should help provide best practice toolkits and expand support for initiatives to implement and disseminate evidence-based interventions.^26^ Researchers modeling the comparative effectiveness of different screening strategies should use empiric data on actual rates of repeat testing over time in simulation models (vs. assuming optimal adherence).

A major strength of this paper is our examination of repeat cancer screening patterns and predictors across three organ types, large, diverse health systems and patient populations, and use of clinically granular EHR data. However, our findings should be interpreted in the context of a few limitations. PROSPR was primarily composed of large integrated health systems, so generalizability to different settings is uncertain. However, the patient populations in PROSPR are representative of the age, gender, and racial/ethnic balance of the US screen eligible population.^6^ We examined testing over multiple screening rounds only for the annual testing regimens used for CRC (stool-based) and lung cancer screening. The study did not include people who started with colonoscopy-based screening, so our results do not reflect the total proportion of people up-to-date with CRC screening. Patterns of repeat testing for less frequent modalities like colonoscopy (every 10 years) and Pap and HPV co-testing (every 3–5 years) would require extremely long follow-up periods. The relatively smaller size of the lung cancer cohort limited power to detect modest associations. Lastly, we did not have information regarding patient preferences or life expectancy that might explain lack of repeat testing in some individuals.

In conclusion, repeat screening for colorectal, cervical, and lung cancer in real-world practice was suboptimal and declined as the number of testing rounds increased. System-level strategies are needed to increase screening adherence given the strong relationship between health system and outcomes.

## Supplementary Information

Below is the link to the electronic supplementary material.

Supplementary Material: Title for eFigure 1: Colorectal Cancer Cohort for “Next Round” Screening Analysis. Legend for eFigure 1: ^1^This group is screening-eligible at the time of index screen (based on age, History of IBD, CRC, adenomatous polyps, colectomy, or proctectomy, and without colonoscopy/lower endo within 10 years or sigmoidoscopy within 5 years) and limited to the first enrollment period. Title for eFigure 2: Cervical Cancer Cohort for “Next Round” Screening Analysis. Legend for eFigure 2: ^1^This group is screening-eligible at the time of index screen (based on age, history of hysterectomy, HPV, HIV, abnormal pap, cervical cancer and limited to the first enrollment period. Title for eFigure 3: Lung Cancer Cohort for “Next Round” Screening Analysis. Legend for eFigure 3: ^1^Eligible for lung cancer screening at the time of index screen (based on age, smoking status, and prior cancer) and limited to the first enrollment period. Title for eFigure 4: Cohort for Colorectal Cancer Screening (CRC) “Annual Screening Consistency” Analysis. Legend for eFigure 4: ^1^This group is screening-eligible at the time of index screen (based on age, History of IBD, CRC, adenomatous polyps, colectomy, or proctectomy, and without colonoscopy/lower endo within 10 years or sigmoidoscopy within 5 years) with at least 15 months of study observation time before cohort exit or exit age, and no “early” tests or diagnoses (less than 9 months after index negative FIT) including FIT, colonography, sigmoidoscopy, colonoscopy or adenoma/CRC diagnosis. Title for eFigure 5: Cohort for Lung Cancer Screening “Annual Screening Consistency” Analysis. Legend for eFigure 5: ^1^Eligible for LCS at the time of index screen (based on age, smoking status, and prior cancer) with at least 15 months of study observation time before cohort exit or exit age, and no“early” tests or diagnoses (less than 9 months after index negative screen) including LDCT, chest CT, or lung cancer diagnosis. (DOCX 118 KB)

## Data Availability

The data that support the findings of this paper are not publicly available. However, information regarding the publicly available dataset for the overall PROSPR consortium is available at: https://healthcaredelivery.cancer.gov/prospr/datashare/.
