# Supplementary material for: Longitudinal Adherence to Screening for Colorectal, Cervical, and Lung Cancer in a US Consortium
Source: J Gen Intern Med. 2025 Oct 7;41(7):1761–70. doi: 10.1007/s11606-025-09835-6 (PMC13176415; doi:10.1007/s11606-025-09835-6)
Supplement: Supplementary file 1 — Supplementary Material: Title for eFigure 1: Colorectal Cancer Cohort for “Next Round” Screening Analysis. Legend for eFigure 1: 1This group is screening-eligible at the time of index screen (based on age, History of IBD, CRC, adenomatous polyps, colectomy, or proctectomy, and without colonoscopy/lower endo within 10 years or sigmoidoscopy within 5 years) and limited to the first enrollment period. Title for eFigure 2: Cervical Cancer Cohort for “Next Round” Screening Analysis. Legend for eFigure 2: 1This group is screening-eligible at the time of index screen (based on age, history of hysterectomy, HPV, HIV, abnormal pap, cervical cancer and limited to the first enrollment period. Title for eFigure 3: Lung Cancer Cohort for “Next Round” Screening Analysis. Legend for eFigure 3: 1Eligible for lung cancer screening at the time of index screen (based on age, smoking status, and prior cancer) and limited to the first enrollment period. Title for eFigure 4: Cohort for Colorectal Cancer Screening (CRC) “Annual Screening Consistency” Analysis. Legend for eFigure 4: 1This group is screening-eligible at the time of index screen (based on age, History of IBD, CRC, adenomatous polyps, colectomy, or proctectomy, and without colonoscopy/lower endo within 10 years or sigmoidoscopy within 5 years) with at least 15 months of study observation time before cohort exit or exit age, and no “early” tests or diagnoses (less than 9 months after index negative FIT) including FIT, colonography, sigmoidoscopy, colonoscopy or adenoma/CRC diagnosis. Title for eFigure 5: Cohort for Lung Cancer Screening “Annual Screening Consistency” Analysis. Legend for eFigure 5: 1Eligible for LCS at the time of index screen (based on age, smoking status, and prior cancer) with at least 15 months of study observation time before cohort exit or exit age, and no“early” tests or diagnoses (less than 9 months after index negative screen) including LDCT, chest CT, or lung cancer diagnosis. (DOCX 118 KB) [file 11606_2025_9835_MOESM1_ESM.docx]

Online Supplement for Halm et al. “Longitudinal Adherence to screening for colorectal, cervical, and lung cancer in a US consortium”

1. eTable 1: Application of the PROSPR cancer screening conceptual model to selecting the study population and operationalizing the repeat screening outcomes across colorectal, cervical and lung cancer
2. eTable 2: Characteristics of patients in the PROSPR three cancer screening cohorts
3. eTable 3: Factors associated with annual screening consistency for colorectal cancer
4. eTable 4: Factors associated with annual screening consistency for lung cancer
5. eFigure 1: Colorectal cancer screening cohort for “Next Round” screening analysis
6. eFigure 2: Cervical cancer screening cohort for “Next Round” screening analysis
7. eFigure 3: Lung cancer screening cohort for “Next Round” screening analysis
8. eFigure 4: Cohort for colorectal cancer screening “Annual Screening Consistency” analysis
9. eFigure 5: Cohort for lung cancer screening “Annual Screening Consistency” analysis

**eTable 1:** Application of the PROSPR cancer screening conceptual model to selecting the study population and operationalizing the repeat screening outcomes across colorectal, cervical and lung cancer

| **Study Design Elements** | **Colorectal Cancer (PRECISE)** | **Cervical Cancer (METRICS)** | **Lung Cancer (LOTUS)** |
| --- | --- | --- | --- |
| **Study Population:**  Age eligible with negative index screen | Adults aged 50-75 years with a negative index fecal immunochemical test (FIT) or guaiac fecal occult blood test (gFOBT) in 2010-2018 and had at least 15 months of subsequent observation time to ascertain repeat testing | Women aged 21-65 years with a negative index Pap in 2010-2016 and had at least 39 months of subsequent observation time or a negative HPV co-test in 2010- 2014 and had at least 63 months of subsequent observation time to ascertain repeat testing | Adults aged 55-80 years with a history of smoking who completed a negative index low dose computed tomography (LDCT) in 2014-2018 and had at least 15 months of subsequent observation time to ascertain repeat testing |
| **Exclusion criteria:**  Ineligible for screening due  to cancer history, or high-risk condition | History of colorectal cancer, adenoma, colectomy or proctectomy, or inflammatory bowel disease | History of cervical cancer, abnormal Pap or positive HPV test, HIV+, or hysterectomy | History of lung cancer |
| **Exclusion criteria:**  Index screening tests with long screening intervals | Colonoscopy in <10 years or sigmoidoscopy in <5 years before index FIT/gFOBT  Negative index colonoscopy (10-year screening interval) | Pap in <3 years or co-test in <5 years before index Pap/Co-test | Not applicable |
| **Exclusion criteria:** Premature screening, diagnostic exam, or cancer diagnosis <9 months after index test | FIT/gFOBT, colonoscopy, sigmoidoscopy, colonography or new adenoma or colorectal cancer diagnosis < 9 months of index test | Pap/HPV test, colposcopy, treatment, or cervical cancer diagnosis < 9 months of index test | LCDT, other chest CT, or new lung cancer diagnosis < 9 months after index test |
| **Eligibility requirements:** Sufficient study observation time to ascertain the next screening test (with 3 months of leeway) | “Next Screen Completed” analyses: continuously enrolled for 15 months after index test  “Annual Screening Consistency” analyses: ≥  2 rounds of study observation time  -Group 1: 30-44 months after index test (0-2 repeat tests)  -Group 2: 45-60 months after index test (0-3 repeat tests) | “Next Screen Completed” analyses: continuously enrolled for 3 years & 3 months  “Annual Screening Consistency” analyses: not analyzed | “Next Screen Completed” analyses: continuously enrolled for 15 months “Annual Screening Consistency” analyses: ≥ 2 rounds of study observation time  -Group 1: 30-44 months after index test (0-2 repeat tests)  -Group 2: 45-60 months after index test time (0-3 repeat tests) |

| **Eligibility requirements: (Continued)** | Cross-overs to colonoscopy were excluded from subsequent rounds given focus on annual FIT testing patterns |  |  |
| --- | --- | --- | --- |
| **“Next Screen Completed” analyses primary outcome:** Next round screening test completion in recommended interval (repeat vs. no repeat screening) | Completion of FIT/gFOBT, colonoscopy, sigmoidoscopy, barium enema, CT colonography, or lower endoscopy not otherwise specified (regardless of indication) 9 to 15 months after negative index stool test | Completion of Pap or co-test (Pap and HPV test) 9-39 months after negative index test | Completion of LCDT or chest CT 9- 15 months after negative index test |
| **“Annual Screening Consistency” analyses primary outcome:** Screening consistency for annual testing modalities over next 2-3 rounds (consistent, inconsistent, no screening) | Screening consistency based on # of FIT/gFOBT tests completed the over next 2-3 rounds  Group 1 (30-44 months of study time):   - No repeat screening = 0 tests in 2 rounds - Inconsistent repeat screening = 1 test in 2 rounds - Consistent repeat screening = 2 tests in 2 rounds   Group 2: 45-60 months of study time   - No repeat screening = 0 tests in 3 rounds - Inconsistent repeat screening = 1 or 2 tests in 3 rounds - Consistent repeat screening = 3 tests in 3 rounds   Cross-overs to colonoscopy were excluded in subsequent rounds given focus on annual FIT testing patterns | Not analyzed | Screening consistency based on the # of LCDT tests completed the over next 2-3 rounds  Group 1 (30-44 months of study time):   - No repeat screening = 0 tests in 2 rounds - Inconsistent repeat screening = 1 test in 2 rounds - Consistent repeat screening   = 2 tests in 2 rounds  Group 2: 45-60 months of study time   - No repeat screening = 0 tests in 3 rounds - Inconsistent repeat screening = 1 or 2 tests in 3 rounds - Consistent repeat screening   = 3 tests in 3 rounds |

**eTable 2:** Characteristics of patients in the PROSPR three cancer screening cohorts

| Characteristic | Colorectal Cancer PRECISE  N= 1,566,347 | Cervical Cancer METRICS  N= 216,344 | Lung Cancer LOTUS N= 6,209 |
| --- | --- | --- | --- |
| Age (years) Mean (SD) | 58.2 (6.7) | 39.4 (12.0) | 64.6 (5.8) |
| Sex  Male Female | 722,116 (46.1%)  844,201 (53.9%) | NA (100%) | 3,347 (53.9%)  2,862 (46.1%) |
| Race/Ethnicity^a^ White  Black Asian  Native Hawaiian/Pacific Islander American Indian/Alaskan Native Hispanic  Multiple, Another, or Unknown | 794,236 (50.7%)  118,605 (7.6%)  222,505 (14.2%)  9,711 (0.6%)  4,629 (0.3%)  352,772 (22.5%)  63,889 (4.1%) | 96.660 (44.7%)  24,710 (11.4%)  15,350 (7.1%)  770 (0.4%)  599 (0.3%)  71,464 (33.0%)  6,791 (3.1%) | 4,350 (70.1%)  915 (14.7%)  235 (3.8%)  30 (0.5%)  15 (0.2%)  266 (4.3%)  398 (6.4%) |
| Body Mass Index (BMI; kg/m^2^) BMI <18.5  BMI 18.5 - < 25  BMI 25 - <30  BMI ≥30  Unknown | 8,352 (0.5%)  267,977 (17.1%)  357,528 (22.8%)  335,787 (21.4%)  596,703 (38.1%) | 3,203 (1.5%)  71,953(33.3%)  62,836 (29.0%)  74,983 (34.7%)  3,369 (1.6%) | 139 (2.2%)  1,508 (24.3%)  2,199 (35.4%)  2,336 (37.6%)  27 (0.4%) |
| Charlson Comorbidity Score 0  1  2  ≥3  Unknown | 996,485 (71.9%)  219,422 (15.8%)  97,531 (7.0%)  72,944 (5.3%)  179,965 (11.5%) | 175,577 (81.2%)  21,888 (10.1%)  7,224 (3.3%)  3,135 (1.5%)  8,520 (3.9%) | 2,194 (35.3%)  1,921 (30.9%)  912 (14.7%)  1,118 (18.0%)  64 (1.0%) |
| Insurance Commercial/Private Medicaid  Medicare  Other Gov. or Uninsured Unknown | 1,140,568 (72.8%)  36,057 (2.3%)  338,187 (21.6%)  31,296 (2.0%)  20,239 (1.3%) | 119,122 (55.1%)  48,948 (22.6%)  3,695 (1.7%)  43,611 (20.2%)  968 (0.5%) | 2,249 (36.2%)  566 (9.1%)  3,158 (50.9%)  10 (0.2%)  226 (3.6%) |
| Year of Index Screen 2010  2011  2012  2013  2014  2015  2016  2017  2018 | 418,625 (26.7%)  221,952 (14.2%)  154,655 (9.9%)  137,338 (8.8%)  150,324 (9.6%)  139,995 (8.9%)  124,538 (8.0%)  121,458 (8.0%)  97,462 (6.2%) | 64,696 (29.9%)  52,226 (24.1%)  36,334 (16.8%)  24,107 (11.1%)  18,042 (8.3%)  12,832 (5.9%)  8,107 (3.8%)  -  - | -  -  -  -  256 (4.1%)  1,030 (16.6%)  1,751 (28.2%)  2,112 (34.0%)  1,060 (17.1%) |
| PCP visit in year prior to index screen Yes  No  Unknown | 1,290,288 (82.4%)  275,752 (17.6%)  307 (0.02%) | 113,547 (52.5%)  26,525 (12.3%)  76,272 (35.3%) | 6,000 (96.6%)  203 (3.3%)  6 (0.1%) |

aAll race/ethnic categories are Non-Hispanic except those included in the Hispanic category. “Multiple” race includes any one selected more than one unique race. Age, insurance, Charlson score, and BMI were are all from the year of index screen, with BMI using last result carried-forward up to 2 years (due to larger quantity of missing data). “Commercial/Private” includes

commercial or high-deductible insurance. “Other Government” insurance includes publicly funded medical assistance programs that are not Medicare or Medicaid. The three different cancer screening cohorts had overlapping but different time periods.

**eTable 3:** Factors associated with annual screening consistency for colorectal cancer

|  | Group 1: 30-44 months  (0 – 2 repeats after index screen) N=692,662 | | Group 2: 45-60 months  (0 – 3 repeats after index screen) N=476,298 | |
| --- | --- | --- | --- | --- |
| Variable | Adjusted Odds Ratio Consistent  Screening | Adjusted Odds Ratio Inconsistent  Screening | Adjusted Odds Ratio Consistent  Screening | Adjusted Odds Ratio  Inconsistent Screening |
| Site |  |  |  |  |
| A | 1.00 (REF) | 1.00 (REF) | 1.00 (REF) | 1.00 (REF) |
| B | 0.12 (0.11-0.12) | 0.77 (0.74-0.79) | 0.10 (0.09-0.11) | 0.61 (0.58-0.64) |
| C | 0.01 (0.01-0.02) | 0.20 (0.18-0.21) | 0.01 (0.01-0.01) | 0.17 (0.16-0.19) |
| D | 0.60 (0.59-0.62) | 0.97 (0.94-1.00) | 0.39 (0.38-0.41) | 0.72 (0.69-0.75) |
| Age (years) | 1.03 (1.03-1.03) | 0.99 (0.99-1.00) | 0.83 (0.82-0.83) | 0.88 (0.87-0.88) |
| Sex |  |  |  |  |
| Male | 1.00 (REF) | 1.00 (REF) | 1.00 (REF) | 1.00 (REF) |
| Female | 0.96 (0.94-0.98) | 1.15 (1.12-1.17) | 1.06 (1.04-1.09) | 1.15 (1.12-1.17) |
| Race/Ethnicity^a^ |  |  |  |  |
| White | 1.00 (REF) | 1.00 (REF) | 1.00 (REF) | 1.00 (REF) |
| Black | 0.81 (0.78-0.83) | 0.92 (0.89-0.94) | 0.74 (0.71-0.77) | 0.88 (0.85-0.91) |
| Asian | 1.04 (1.02-1.07) | 1.01 (0.98-1.04) | 1.13 (1.09-1.17) | 1.06 (1.03-1.10) |
| Native Hawaiian/Pacific Islander | 0.78 (0.70-0.86) | 0.92 (0.83-1.02) | 0.74 (0.65-0.83) | 0.83 (0.73-0.93) |
| American Indian/Alaskan Native | 0.70 (0.61-0.80) | 0.83 (0.72-0.96) | 0.71 (0.58-0.86) | 0.81 (0.68-0.97) |
| Hispanic | 0.85 (0.84-0.87) | 1.03 (1.01-1.05) | 0.79 (0.77-0.81) | 0.97 (0.94-0.99) |
| Multiple/Another | 0.67 (0.64-0.70) | 0.76 (0.73-0.80) | 0.66 (0.63-0.70) | 0.78 (0.74-0.82) |
| Body Mass Index (BMI) |  |  |  |  |
| BMI ≥18.5 - < 25 | 1.00 (REF) | 1.00 (REF) | 1.00 (REF) | 1.00 (REF) |
| BMI ≥25 - <30 | 0.92 (0.90-0.95) | 1.02 (0.99-1.05) | 0.85 (0.81-0.88) | 0.96 (0.92-1.00) |
| BMI ≥30 | 0.81 (0.78-0.83) | 0.97 (0.94-0.99) | 0.70 (0.67-0.73) | 0.89 (0.86-0.93) |
| BMI <18.5 | 0.80 (0.71-0.89) | 0.82 (0.73-0.92) | 0.85 (0.71-1.03) | 0.85 (0.70-1.02) |
| Unknown | 0.48 (0.47-0.50) | 0.71 (0.68-0.73) | 0.54 (0.51-0.56) | 0.80 (0.77-0.84) |
| Charlson Comorbidity Score |  |  |  |  |
| 0 | 1.00 (REF) | 1.00 (REF) | 1.00 (REF) | 1.00 (REF) |
| 1 | 1.02 (1.00-1.04) | 1.06 (1.04-1.09) | 1.07 (1.03-1.10) | 1.14 (1.11-1.18) |
| 2 | 1.04 (1.01-1.07) | 1.09 (1.06-1.13) | 1.08 (1.03-1.14) | 1.15 (1.09-1.21) |
| ≥3 | 0.84 (0.81-0.87) | 1.02 (0.98-1.05) | 0.92 (0.86-0.99) | 1.06 (0.99-1.13) |
| Smoking Status |  |  |  |  |
| Never Smoked or Unknown | 1.00 (REF) | 1.00 (REF) | 1.00 (REF) | 1.00 (REF) |
| Ever Smoked | 0.81 (0.80-0.83) | 0.92 (0.91-0.94) | 0.77 (0.75-0.79) | 0.90 (0.88-0.92) |
| Insurance |  |  |  |  |
| Commercial/Private | 1.00 (REF) | 1.00 (REF) | 1.00 (REF) | 1.00 (REF) |
| Medicaid | 0.96 (0.90-1.02) | 0.95 (0.89-1.01) | 0.81 (0.75-0.88) | 0.94 (0.87-1.02) |
| Medicare | 1.29 (1.26-1.32) | 1.16 (1.13-1.19) | 1.16 (1.09-1.24) | 1.09 (1.02-1.16) |
| Other Govt. or Uninsured | 1.29 (1.16-1.42) | 1.07 (0.99-1.16) | 1.24 (1.05-1.46) | 1.03 (0.92-1.14) |
| PCP visit year prior to index |  |  |  |  |
| Yes | 1.00 (REF) | 1.00 (REF) | 1.00 (REF) | 1.00 (REF) |
| No | 1.05 (1.02-1.07) | 1.00 (0.98-1.03) | 1.11 (1.08-1.14) | 1.00 (0.97-1.03) |

The adjusted odds of consistent and inconsistent screening are compared to the ‘no repeat screening’ reference group. aAll race/ethnic categories are non-Hispanic except those included in the Hispanic category. “Multiple” race includes any one selected more than one unique race. Age, insurance, Charlson score, smoking status, and BMI are from the year of index screen, with BMI also using last result carried-forward up to 2 years (due to larger quantity of missing data). “Commercial/Private” includes commercial or high-deductible insurance. “Other Government” insurance includes publicly funded medical assistance programs that are not Medicare or Medicaid.

**eTable 4:** Factors associated with annual screening consistency for lung cancer

|  | Group1: 30-44 months  (0 – 2 repeats after index screen) N=2,121 | | Group 2: 45-60 months  (0 – 3 repeats after index screen) N=994 | |
| --- | --- | --- | --- | --- |
| Variable | Adjusted Odds Ratio Consistent Screening | Adjusted Odds Ratio Inconsistent Screening | Adjusted Odds Ratio Consistent Screening | Adjusted Odds Ratio Inconsistent Screening |
| Site |  |  |  |  |
| A | 1.00 (REF) | 1.00 (REF) | 1.00 (REF) | 1.00 (REF) |
| B | 1.07 (0.49-2.32) | 0.41 (0.17-1.01) | 1.60 (0.52-4.91) | 1.19 (0.38-3.80) |
| C | 0.05 (0.03-0.07) | 0.25 (0.17-0.38) | 0.07 (0.03-0.14) | 0.46 (0.25-0.88) |
| D | 0.16 (0.10-0.28) | 0.35 (0.21-0.61) | 0.22 (0.08-0.60) | 0.65 (0.25-1.71) |
| E | 0.04 (0.02-0.06) | 0.28 (0.18-0.42) | 0.02 (0.01-0.05) | 0.18 (0.10-0.33) |
| Age (years) | 1.03 (1.00-1.06) | 1.03 (1.00-1.06) | 1.05 (0.99-1.11) | 1.07 (1.02-1.12) |
| Sex |  |  |  |  |
| Male | 1.00 (REF) | 1.00 (REF) | 1.00 (REF) | 1.00 (REF) |
| Female | 1.07 (0.81-1.40) | 1.03 (0.80-1.33) | 0.89 (0.56-1.43) | 1.20 (0.78-1.86) |
| Race/Ethnicity^a^ |  |  |  |  |
| White | 1.00 (REF) | 1.00 (REF) | 1.00 (REF) | 1.00 (REF) |
| Black | 0.63 (0.42-0.93) | 0.81 (0.59-1.11) | 0.63 (0.29-1.40) | 1.15 (0.64-2.07) |
| Asian | 1.74 (0.66-4.61) | 1.43 (0.53-3.91) | 0.84 (0.21-3.44) | 0.60 (0.14-2.61) |
| Native Hawaiian/Pacific Islander | 1.61 (0.15-17.08) | 1.56 (0.13-18.15) | 0.19 (0.03-1.34) | 0.06 (0.00-0.71) |
| Hispanic | 0.88 (0.39-2.01) | 0.85 (0.36-1.98) | 0.66 (0.20-2.15) | 0.84 (0.26-2.76) |
| AI, AN, Multiple or Another | 0.84 (0.49-1.46) | 0.82 (0.48-1.39) | 0.84 (0.21-3.44) | 0.60 (0.14-2.61) |
| Body Mass Index (BMI) |  |  |  |  |
| BMI ≥18.5 - < 25 | 1.00 (REF) | 1.00 (REF) | 1.00 (REF) | 1.00 (REF) |
| BMI ≥25 - <30 | 1.18 (0.82-1.69) | 1.26 (0.90-1.77) | 0.92 (0.51-1.66) | 0.86 (0.50-1.50) |
| BMI ≥30 | 0.83 (0.58-1.19) | 1.05 (0.75-1.46) | 1.42 (0.76-2.66) | 1.24 (0.70-2.21) |
| BMI <18.5 | 0.80 (0.31-2.09) | 0.91 (0.38-2.20) | 0.34 (0.08-1.44) | 0.51 (0.15-1.77) |
| Charlson Comorbidity Score |  |  |  |  |
| 0 | 1.00 (REF) | 1.00 (REF) | 1.00 (REF) | 1.00 (REF) |
| 1 | 1.85 (1.33-2.56) | 1.43 (1.05-1.93) | 0.97 (0.56-1.68) | 0.79 (0.48-1.32) |
| 2 | 2.54 (1.66-3.89) | 1.48 (0.99-2.22) | 1.05 (0.50-2.17) | 0.98 (0.50-1.93) |
| ≥3 | 1.34 (0.88-2.04) | 1.71 (1.16-2.51) | 1.43 (0.65-3.13) | 1.11 (0.53-2.32) |
| Smoking Status |  |  |  |  |
| Former Smoker | 1.00 (REF) | 1.00 (REF) | 1.00 (REF) | 1.00 (REF) |
| Current Smoker | 0.70 (0.53-0.93) | 1.04 (0.80-1.36) | 2.21 (1.37-3.55) | 1.94 (1.25-3.01) |
| Insurance |  |  |  |  |
| Commercial/Private | 1.00 (REF) | 1.00 (REF) | 1.00 (REF) | 1.00 (REF) |
| Medicare | 1.01 (0.70-1.48) | 0.90 (0.64-1.26) | 0.74 (0.39-1.40) | 0.76 (0.43-1.33) |
| Medicaid, Uninsured, Other | 0.71 (0.44-1.15) | 0.96 (0.62-1.49) | 0.69 (0.29-1.63) | 0.89 (0.43-1.84) |
| PCP visit year prior to index |  |  |  |  |
| Yes | 1.00 (REF) | 1.00 (REF) | 1.00 (REF) | 1.00 (REF) |
| No | 2.35 (1.02-5.41) | 2.01 (0.90-4.47) | 1.73 (0.37-8.06) | 0.82 (0.19-3.50) |

aAll race/ethnic categories are non-Hispanic except those included in the Hispanic category. “Multiple” race includes any one selected more than one unique race. AI = American Indian, and AN = Alaskan Native. Due to smaller sample sizes, we combined AI, AN, with the Multiple/Another category for the lung cancer screening analyses. Age, insurance, Charlson score, smoking status, and BMI were from the year of index screen, with smoking status and BMI also using last result carried- forward up to 2 years. Insurance category “Medicaid, Uninsured, Other” includes Medicaid, Other Government Insurance, “Other”, uninsured, or medical assistance. “Commercial/Private” includes commercial or high-deductible insurance.

Index screen & at least 15 months of study observation time

N = 1,772,441

- Less than 15 months follow-up between index screen and cohort exit: N = 391,803

eFigure 1

Colorectal cancer screening-eligible with a negative index FIT1

N = 2,164,244

Final repeat CRC cancer screening-eligible cohort N = 1,566,347

-Early (less than 9 months after index negative FIT) FIT, colonography, sigmoidoscopy, colonoscopy or adenoma/cancer dx: N= 177,841

Index screen, at least 15 months of study observation time, & age eligible at the end of follow-up period

N = 1,744,188

- Reached screening-exit age during follow-up period; N = 28,253

Cervical cancer screening-eligible with a negative index screen1

N = 438,046

Index screen & at least 3 years & 3 months of study observation time N = 235,267

- Less than 39 months follow-up between index screen and cohort exit: N = 202,779

Index screen, at least 3 years & 3 months of study observation time, & age eligible at the end of study period N = 223,817

- Reached screening-exit age during follow-up period: N = 11,450

Final repeat cervical cancer screening- eligible cohort

N = 216,344

Early (less than 9 months after index negative screen) test, colposcopy or treatment, abnormal result, cancer dx: N= 7,473

eFigure 2

Lung cancer screening-eligible with a negative index screen1

N = 10,814

Index screen & at least 15 months of study observation time N = 6,746

- Less than 15 months follow-up between index screen and cohort exit: N = 4,068

Index screen, at least 15 months of study observation time, & age eligible at the end of follow-up period

N = 6,698

- Reached screening-exit age during follow-up time: N = 48

Final repeat lung cancer screening-eligible cohort N = 6,209

-Early (less than 9 months after index negative screen) test or lung cancer diagnosis: N= 489

eFigure 3

CRC screening cohort1

N = 1,566,347

**CRC Group 2**🡪 45-60 months of study observation time between index screen and cohort exit, aging out, colonoscopy, or CRC dx and negative FIT in Round 2

N = 476,298

**CRC Group 1:**

30-44 months of study observation time

N = 692,662

CRC screening consistency analysis eligible cohort

N = 1,168,960

- Positive FIT round 1 (before 15 months): N= 28,454
- Reached screening-exit age before 30 months: N = 24,439
- Colonoscopy, colectomy or other colorectal procedure before 30 months: N = 71,819
- CRC diagnosis before 30 months: N = 132

Index screen and 30 months of study observation time

N = 1,293,804

- Less than 30 months follow-up between index screen and cohort exit (enrollment): N = 272,543

eFigure 4

Lung cancer screening cohort1

N = 6,209

Index screen and 30 months of follow-up:

N = 3,276

- Less than 30 months follow-up between index screen and cohort exit (enrollment): N = 2,933

- Positive LCS test in Round 1: N = 122
- Reached screening-exit age before 30 months: N = 12
- Lung cancer diagnosis before 30 months: N = 27

**Lung Cancer Group 1:**

Lung cancer screening consistency analysis eligible cohort

N = 3,115

30-44 months of study observation time N = 2,121

**Lung Cancer Group 2**🡪 45-60 months of study observation time between index screen and cohort exit, aging out, or lung cancer dx AND negative LCS in Round 2: N = 994

eFigure 5
